# Disease avoidance in the time of COVID-19: The behavioral immune system is associated with concern and preventative health behaviors

**DOI:** 10.1371/journal.pone.0238015

**Published:** 2020-08-20

**Authors:** Natalie J. Shook, Barış Sevi, Jerin Lee, Benjamin Oosterhoff, Holly N. Fitzgerald

**Affiliations:** 1 University of Connecticut, Storrs, Connecticut, United States of America; 2 Montana State University, Bozeman, Montana, United States of America; National Center for Global Health and Medicine, JAPAN

## Abstract

The coronavirus disease 2019 (COVID-19) poses a serious global health threat. Without a vaccine, behavior change is the most effective means of reducing disease transmission. Identifying psychological factors that may encourage engagement in preventative health behaviors is crucial. The behavioral immune system (BIS) represents a set of psychological processes thought to promote health by encouraging disease avoidance behaviors. This study examined whether individual differences in BIS reactivity (germ aversion, pathogen disgust sensitivity) were associated with concern about COVID-19 and engagement in recommended preventative health behaviors (social distancing, handwashing, cleaning/disinfecting, avoiding touching face, wearing facemasks). From March 20 to 23, 2020, a US national sample (*N* = 1019) completed an online survey. Germ aversion and pathogen disgust sensitivity were the two variables most consistently associated with COVID-19 concern and preventative health behaviors, while accounting for demographic, health, and psychosocial covariates. Findings have implications for the development of interventions intended to increase preventative health behaviors.

## Introduction

In December 2019, there was an outbreak of a novel coronavirus originating in Wuhan, China. The virus and its resultant infection, coronavirus disease 2019 (COVID-19), spread rapidly across the globe, leading the World Health Organization (WHO) to label the outbreak a pandemic on March 11, 2020 [1]. As of May 18, 2020, there were over 4.5 million confirmed cases of COVID-19 worldwide and over 300,000 deaths [2]. In order to decrease transmission of the virus and reduce likelihood of illness, health organizations recommended a number of preventative health behaviors, such as washing hands frequently and thoroughly, avoiding touching one’s face, disinfecting and cleaning frequently touched surfaces, engaging in social distancing (i.e., avoiding close contact with others), and wearing facemasks [3, 4]. On March 13, 2020, the US declared COVID-19 a national emergency [5], which prompted many—*but not all*—US citizens to follow health organization guidelines and drastically change their everyday behaviors in order to avoid contracting and spreading COVID-19. With no vaccine currently available to protect against COVID-19, behavior change is the most effective means of disease prevention [6]. Thus, it is crucial to identify psychological factors that account for differences in compliance with health recommendations in order to create efficacious strategies to encourage behavior change and limit the spread of the disease, or future infectious diseases.

A set of psychological factors that may be particularly relevant for understanding responses to COVID-19 comes from theory on the behavioral immune system (BIS) [7]. Although the physiological immune system is generally effective in combating pathogens (e.g., coronavirus) and infection, it is limited by being both reactive and metabolically costly [8]. The BIS is thought to have evolved as a complementary “first line of defense” against pathogen threat [7]. Specifically, the BIS is comprised of affective and cognitive processes (e.g., germ aversion and disgust sensitivity) that facilitate the identification of potential pathogen sources and encourage avoidance behavior, thus reducing the likelihood of infection and conserving the physiological immune system [9].

The BIS is proposed to be adaptively successful, because it is characterized by “functional flexibility” [9, 10]. That is, components of the BIS can be activated by environmental cues and there are trait-level differences in BIS reactivity [9, 10]. Indeed, individuals reliably vary in disgust sensitivity, or how easily one becomes disgusted and the strength of their disgust response [11, 12], and germ aversion, or dislike of situations involving the potential for contact with pathogens [13]. Those higher in BIS reactivity should be more sensitive to pathogen threats and more avoidant of potential contamination. As such, individuals higher in disgust sensitivity and germ aversion should be more concerned with the COVID-19 pandemic and more likely to engage in preventative health behaviors to avoid the disease. Some initial evidence supports this proposition, such that greater BIS reactivity has been linked to behavioral intentions and past efforts to follow COVID-19 health guidelines [14] and support for travel bans on Asian countries [15].

The BIS presents a promising set of psychological processes that could be targeted and potentially modified to encourage behavior change. However, examining links between the BIS and COVID-19 responses requires certain methodological considerations. There are a number of demographic, health, social, and personality factors that may influence concern about COVID-19 and engagement in preventative health behaviors. For instance, greater religiosity and conscientiousness have been associated with engaging in more health promotive behaviors (e.g., more physical activity, less alcohol consumption; [16, 17]), which may extend to COVID-19 preventative health behaviors. Neuroticism has been associated with more attention to health concerns and physical symptoms [18], but less health promotive behavior [17]. Political orientation has specifically been associated with differential responses to COVID-19, with Democrats reporting more concern and greater engagement in preventative health behaviors than Republicans [19]. Each of these factors have also been connected with BIS indicators [20, 21]. Thus, it is important to account for a wide range of demographic and psychosocial characteristics when examining links between individual differences in the BIS and responses to COVID-19.

The goal of the present study was to determine whether BIS indicators (germ aversion, pathogen disgust sensitivity) were associated with COVID-19 concern and engagement in recommended preventative health behaviors. Although germ aversion and pathogen disgust sensitivity are related [22], they represent distinct constructs and components of the BIS. We chose to include both constructs to test the generalizability of findings and assess the unique variance accounted for by each construct. We hypothesized that those who were higher in germ aversion and pathogen disgust sensitivity would be more concerned with COVID-19 and would more frequently engage in social distancing, avoidance of face touching, handwashing, cleaning or disinfecting commonly used surfaces, and wearing a facemask. Of note, at the time of data collection, health organizations did not recommend wearing facemasks by the general public, except if an individual had COVID-19 [23]. To isolate these associations, we accounted for a wide variety of demographic, health, social, and personality characteristics.

## Method

### Participants and procedure

Data were collected on March 20 and 23, 2020, from a nationally representative sample of 1023 individuals residing in the US, recruited through the panel provider Qualtrics for a larger longitudinal study about the effects of COVID-19. Sample size was determined based on Monte Carlo simulations (*N* = 10,000) of the most conservative models for the data analysis plan associated with the larger longitudinal project. A minimum sample of 500 was estimated to provide sufficient power (>95%) to detect anticipated effects (β = .15 to .20) based on previous BIS research assuming α = .05. To account for missing or unusable data, we aimed to recruit a panel of at least 1000 US individuals. The current study used the first wave of data from the longitudinal study. Four participants were excluded from analyses due to problematic response patterns (e.g., gibberish open-ended responses, straight line responses to close-ended measures). The final sample was compromised of 1019 participants (514 female; *M*_age_ = 46.33 years, *SD*_age_ = 16.57, range: 18 to 85 years; 77.3% White; *Mdn*_education_ = College graduate; *Mdn*_income_ = $70,000–$79,999; see Table 1 for a full description of the sample).

**Table 1:** Descriptive statistics for all study variables.

Measure	*M* (*n*)	*SD* (%)	Min.	Max.
**Demographics and Health**				
Age	46.32	16.57	18	85
18–64	765	75.9%		
Gender				
Female	514	50.8%	–	–
Male	497	49.1%	–	–
Other	1	0.1%	–	–
Not reported	7	0.7%	–	–
Ethnicity/Race				
White	788	77.3%	–	–
Latinx/Hispanic	38	3.7%	–	–
Black	64	6.3%	–	–
Asian	73	7.2%	–	–
Native American	4	0.4%	–	–
Other	6	0.6%		
Multi	35	3.4%	–	–
Not reported	11	1.1%		
Income				
Less than $10,000	36	3.5%	–	–
$10,000–$19,999	32	3.1%	–	–
$20,000–$29,999	58	5.7%	–	–
$30,000–$39,999	81	7.9%	–	–
$40,000–$49,999	86	8.4%	–	–
$50,000–$59,999	88	8.6%	–	–
$60,000–$69,999	85	8.3%	–	–
$70,000–$79,999	94	9.2%	–	–
$80,000–$89,999	59	5.8%	–	–
$90,000–$99,999	59	5.8%	–	–
$100,000–$149,999	201	19.7%	–	–
More than $150,000	131	12.9%	–	–
Not reported	9	0.9%	–	–
Education				
Less than/some high school	11	1.1%	–	–
GED/high school equivalency	19	1.9%	–	–
High school graduate	107	10.5%	–	–
Vocation/trade school	27	2.6%	–	–
Some college	151	14.8%	–	–
Associate’s 2-year degree	86	8.4%	–	–
College graduate	343	33.7%	–	–
Graduate studies/professional degree	266	26.1%	–	–
Not reported	9	0.9%	–	–
Residence Town Size				
Rural (unincorporated)	104	10.2%	–	–
Small town (village or town)	102	10%	–	–
Suburban (metropolitan area of a large city)	434	42.6%	–	–
Small city (population <30,000)	52	5.1%	–	–
Medium-sized city (population 30,000–100,000)	89	8.7%	–	–
Large city (population >100,000)	229	22.5%	–	–
Not reported	9	0.9%	–	–
Work in Healthcare (Yes)	89	8.7%	–	–
Self COVID-19 risk status (high)	417	40.9%	–	–
Family COVID-19 risk status (high)	560	55.0%	–	–
Illness Recency	2.44	1.66	1	7
Perceived Health	3.62	0.89	1	5
COVID-19 Status (Indicated never had)	838	82.2%	–	–
Perceived Infectability	3.50	1.08	1	7
**Psychosocial Variables**				
Religiosity	4.73	3.60	0	10
Political Orientation	2.96	1.15	1	5
Extraversion	2.97	0.95	1	5
Agreeableness	3.47	0.86	1	5
Conscientiousness	3.84	0.89	1	5
Neuroticism	2.70	1.00	1	5
Openness	3.25	0.85	1	5
COVID-19 Concern^	0.00	0.72	-2.51	1.07
Social Distancing^	0.00	0.68	-1.37	1.60
Avoid Touching Face	3.55	1.92	1	6
Wearing Antiviral Facemask	1.77	1.42	1	6
Handwashing	3.99	1.38	1	6
Cleaning/Disinfecting	2.90	1.50	1	6
**BIS Indicators**				
Germ Aversion	4.68	0.96	1	7
Pathogen Disgust	4.29	1.11	0	6

^Items were standardized before creating composite variables.

This project was approved by the University of Connecticut IRB (Protocol #L20-0018). Participants provided electronic consent before completing the study. After electronically agreeing to be part of the study, participants completed the primary study measures and other questionnaires in a random order, except for perceived health, illness recency, health history, and demographics which appeared last (see S1 Appendix for all measures). Upon completion, participants were given monetary compensation in an amount established by the panel provider. The online survey took approximately 27 minutes to complete.

### Measures

#### BIS indicators

The 15-item Perceived Vulnerability to Disease Questionnaire [13] is an individual difference measure that consists of two subscales: Germ Aversion and Perceived Infectability. The Germ Aversion subscale includes eight items and measures individual discomfort with situations that imply high likelihood of pathogen transmission (e.g., “It really bothers me when people sneeze without covering their mouths”; α = .70). Germ aversion is a component of the BIS. The Perceived Infectability subscale includes seven items and measures perceived personal susceptibility to infectious disease (e.g., “I am more likely than the people around me to catch an infectious disease”; α = .79). Although involving some level of subjective assessment, the perceived infectability subscale does not necessarily assess psychological disease avoidance processes; rather, responses to the items are in large part based on health history and biological susceptibility to infection [24, 25]. As previous likelihood of contracting infectious disease may influence COVID-19 concern and preventative health behaviors, we included perceived infectability as a covariate in the primary analyses. All ratings of items were made on a scale ranging from 1 (“Strongly Disagree”) to 7 (“Strongly Agree”). A composite score for each subscale was created by taking the average of the items. Higher scores reflect greater germ aversion or perceived infectability.

The 7-item pathogen disgust subscale from the Three Domains of Disgust Scale [12] was used to assess individual differences in disgust sensitivity specifically related to pathogens. Participants rated how disgusting they found each item (e.g. “stepping on dog poop”) on a 7-point scale from 0 (“not disgusting at all”) to 6 (“extremely disgusting”). A composite variable was created by taking the average of the items (α = .85). Higher scores reflect greater pathogen disgust sensitivity.

#### COVID-19 concern

At the time of data collection, there were no existing, validated measures of COVID-19 concern. We developed six items to assess the degree to which participants were concerned about COVID-19. One item explicitly asked participants to indicate how concerned they were about COVID-19 on a 5-point scale from 1 (“Not at all concerned”) to 5 (“Very concerned”). For the other five items, participants indicated their agreement with statements regarding the seriousness of the coronavirus (e.g., “The coronavirus is just the flu or a common cold,” “People are not doing enough to prevent the spread of the coronavirus”) on a scale 1 (“Strongly Disagree”) to 6 (“Strongly Agree”). Necessary items were reverse scored, and all items were standardized. A composite measure was created by taking the average of the standardized items (α = .81). Higher scores indicate more concern towards COVID-19.

#### Preventative health behaviors

Participants were asked to indicate how frequently they had engaged in 11 behaviors in the past week on a scale from 1 (“Not at all”) to 6 (“Multiple times a day”). A single item assessed the extent to which participants avoided touching their faces. A single item assessed how often participants washed their hands for at least 20 seconds. A single item assessed frequency of wearing an antiviral mask.

Three items assessed frequency of disinfecting and cleaning frequently touched surfaces (i.e., “Clean and disinfect surfaces in your home with antibacterial wipes,” “Clean your laptop,” and “Clean your mobile phone”). A composite score was created by taking the average of the items (α = .86). Higher scores indicated engaging in each behavior more frequently.

Five items assessed the extent to which participants avoided contact with others (e.g., “Avoid shaking someone’s hand for greeting,” “Avoid going to school/job”). Participants were also asked to indicate the extent to which they were engaging in social distancing on a scale from 1 (“Not at all”) to 5 (“A great deal”). All six items were standardized and a composite measure was created by taking the average of the standardized items (α = .76). Higher scores indicate more social distancing.

#### Personality

A 10-item short version of the Big Five Inventory (BFI-10) [26] was used to assess the big five personality characteristics. Each personality trait was assessed with two items. Participants rated their agreement on a scale from 1 (“Disagree Strongly”) to 5 (“Agree Strongly”) to statements for openness to experience (e.g., “has an active imagination”; *r* = .07), conscientiousness (e.g., “does a thorough job”; *r* = .37), neuroticism (e.g., “gets nervous easily”; *r* = .39), agreeableness (e.g., “is generally trusting”; *r* = .18), and extraversion (e.g., “is outgoing, sociable”; *r* = .28). A composite score for each personality characteristic was created by taking the average of the items. Higher values indicate stronger identification with that trait.

#### COVID-19 infection status

Participants indicated if they thought they have or had COVID-19 by indicating as “yes”, “maybe”, or “no”. For the analyses, the variable was dichotomized. Responses of “yes” or “maybe” were coded as 1, and “no” was coded as 0.

#### Perceived health

Participants rated their overall health in general on a scale from 1 (“Poor”) to 5 (“Excellent”).

#### Recent illness

Illness recency was assessed by asking the participants their agreement with four statements [27]. Using a scale from 1 (“strongly disagree”) to 7 (“strongly agree”), participants indicated their agreement to statements, such as “Over the past couple days, I have not been feeling well”. A composite score was created by taking the average of the items. Higher scores indicate more recent illness (α = .93).

#### Health history

Participants were presented with 46 medical conditions and were asked to indicate whether they and/or a family member (“e.g., your mother, father, sister, brother, aunt, uncle, etc.”) had each condition. Participants were also asked to indicate if they were taking any immunosuppressive medication and for females if they were pregnant. According to the Centers for Disease Control and Prevention (CDC), some individuals are at higher risk for severe illness from COVID-19 [28]. Twenty-two of the medical conditions in the health history questionnaire have been identified by the CDC as placing individuals at risk for severe complications from COVID-19 (e.g., diabetes, dialysis/transplant, cirrhosis), as well as pregnancy and immunosuppressive medication. From this information, a dichotomous variable indicating risk of complications from COVID-19 was created. If participants indicated they had at least one of the medical conditions identified by the CDC, were taking immunosuppressive medication, or were pregnant, they were coded 1 as a participant with high risk of complication from COVID-19. Participants who did not meet any of these criteria were coded 0.

Based on health history information, a variable was also created to indicate whether participants had a family member at risk for complications from COVID-19. If participants indicated that a family member had at least one of the conditions identified by the CDC, they were coded 1 as a participant with a family member at high risk for complications from COVID-19. Participants who had no family members with any of the conditions were coded 0.

#### Political orientation

Participants indicated their political orientation on a 5-point scale from 1 (“Very conservative”) to 5 (“Very liberal”).

#### Religiosity

Participants indicated the extent to which they were religious on an 11-point scale from 0 (“Not at all religious”) to 10 (“Extremely religious”).

#### Demographics

Participants reported their age, race, sex, education level, annual family income, size of town they lived in, and if they were working in a healthcare field.

## Results

### Correlations

Means and standard deviations for all primary study variables are presented in Table 1. Bivariate correlations were estimated between the individual characteristics (i.e., demographics, health, social, personality, and BIS indicators) and concern about COVID-19, as well as the preventative health behaviors (see Table 2). Respondents who were older, White, female, more highly educated, at high risk for complications from COVID-19, had a family member at high risk for complications from COVID-19, had not recently been ill, never had COVID-19, were less religious, were more liberal, were more agreeable, conscientious, or open to experience, and were higher in perceived infectability, germ aversion, or pathogen disgust sensitivity reported greater COVID-19 concern.

**Table 2:** Bivariate correlations between respondent characteristics and COVID-19 concern and preventative health behaviors.

	COVID-19 Concern	Social Distancing	Avoid Touching Face	Wearing Facemask	Hand Washing	Cleaning/Disinfecting
Age	0.19***	-0.07*	-0.06	-0.23***	0.11***	-0.15***
Race	-0.07*	0.03	0.04	0.11***	-0.07*	0.07*
Sex	0.11***	-0.01	0.06	-0.17***	0.17***	0.01
Education	0.10**	0.12***	0.09**	0.01	0.02	-0.02
Income	0.04	0.13***	0.08*	0.07*	0.04	0.06
Hometown	-0.01	0.06	0.08*	0.16***	-0.02	0.07*
Work in Healthcare	-0.06	0.05	0.05	0.16***	-0.01	0.07*
Risk Status (Self)	0.10**	0.09*	-0.01	0.10**	0.02	0.05
Risk Status (Family)	0.11***	-0.01	0.01	-0.09**	0.09**	-0.10**
Illness Recency	-0.17***	0.24***	0.05	0.44***	-0.22***	0.22***
Perceived Health	-0.06	0.09**	0.08*	0.09**	0.09**	0.09**
COVID-19 Status	-0.06*	0.08**	0.05	0.17***	-0.07*	0.06*
Perceived Infectability	0.08**	0.17***	0.11***	0.22***	-0.10***	0.15***
Religiosity	-0.12***	0.21***	0.08*	0.24***	-0.06	0.22***
Political Orientation	0.23***	0.03	0.10***	-0.05	0.08*	0.03
Extraversion	0.04	0.14***	0.08*	0.01	0.04	0.15***
Agreeableness	0.11***	-0.01	0.02	-0.13***	0.11***	-0.01
Conscientiousness	0.20***	-0.01	0.10***	-0.24***	0.25***	0.03
Neuroticism	0.02	0.03	-0.05	0.04	-0.08*	0.01
Openness	0.14***	0.03	0.01	-0.09**	0.08**	0.01
Germ Aversion	0.25***	0.20***	0.27***	-0.07*	0.29***	0.21***
Pathogen Disgust	0.18***	0.22***	0.15***	0.06	0.24***	0.22***
COVID-19 Concern	--	0.24***	0.27***	-0.13***	0.27***	0.10***
Social Distancing	0.24***	--	0.47***	0.44***	0.20***	0.52***
Avoid Touching Face	0.27***	0.47***	--	0.22***	0.35***	0.41***
Wearing Facemask	-0.13***	0.44***	0.22***	--	-0.06	0.43***
Hand Washing	0.27***	0.20***	0.35***	-0.06	--	0.22***
Cleaning/Disinfecting	0.10***	0.52***	0.41***	0.43***	0.22***	--

*p < .05.

**p < .01.

***p ≤ .001. Race was coded: 1 = Not White, 0 = White. Sex was coded: 1 = female, 0 = male. Work in Healthcare was coded: 1 = yes, 0 = no. Risk Status was coded: 1 = high risk, 0 = not high risk. COVID-19 Status was coded: 1 = yes/maybe, 0 = no.

In general, younger age, higher income, more populated location of residence, more recent illness, better perceived health, having/had COVID-19, greater religiosity, greater extraversion, greater conscientiousness, greater perceived infectability, greater germ aversion, and greater pathogen disgust sensitivity were associated with engaging in most (at least three) of the preventative health behaviors more frequently. Of note, however, germ aversion and conscientiousness were negatively associated with wearing an antiviral facemask. Also, younger age, more recent illness, having/had COVID-19, and greater perceived infectability were associated with less frequent handwashing. Greater concern about COVID-19 was associated with more frequent engagement in all of the preventative health behaviors, except for wearing an antiviral facemask. Concern about COVID-19 was negatively associated with wearing an antiviral facemask.

### Regression analyses

To determine whether BIS indicators were uniquely associated with concern about COVID-19 and preventative health behaviors, a series of regression analyses were conducted. BIS indicators (germ aversion and pathogen disgust sensitivity) were entered as primary predictors. Concern about COVID-19 and preventative health behaviors (social distancing, avoidance of face touching, wearing a facemask, hand washing, cleaning/disinfecting) were entered as primary outcomes. Demographic and health characteristics (i.e., age, race, sex, education, income, size of hometown, whether or not one works in healthcare, one’s risk of severe illness from COVID-19, family’s risk of severe illness from COVID-19, illness recency, perceived health, perceived infectability, and history of COVID-19 infection), religiosity, political orientation, and personality (extraversion, openness, conscientiousness, neuroticism, agreeableness) were entered as covariates. For analyses with preventative health behaviors as outcomes, COVID-19 concern was also entered as a covariate. Multicollinearity was checked and found not to be a problem (all VIF < 5, Tolerance > 0.20). Standardized estimates from regression analyses are presented in Table 3 (see S1–S6 Tables for unstandardized estimates, standard errors, and confidence intervals for all models).

**Table 3:** Regression model results for COVID-19 concern and preventative health behaviors.

	COVID-19 Concern	Social Distancing	Avoid Touching Face	Wearing Facemask	Hand Washing	Cleaning/Disinfecting
	*β*	*β*	*β*	*β*	*β*	*β*
*Demographic and Health*						
Age	0.12***	-0.07	-0.07	-0.11**	0.00	-0.14***
Race	-0.02	0.01	0.02	0.01	-0.04	0.01
Sex	0.02	-0.03	0.02	-0.10***	0.10***	-0.01
Education	0.07*	0.05	0.04	-0.03	-0.04	-0.06
Income	0.02	0.07*	0.05	0.05	0.03	0.07*
Hometown	0.01	0.00	0.04	0.08*	0.03	0.02
Work in Healthcare	-0.03	-0.01	0.03	0.05	0.02	0.01
Risk Status (Self)	0.06	0.05	-0.03	0.11***	0.05	0.08*
Risk Status (Family)	0.04	-0.04	0.02	-0.06*	0.03	-0.10***
Illness Recency	-0.13***	0.21***	0.05	0.31***	-0.12***	0.19***
Perceived Health	-0.01	0.08*	0.05	0.13***	0.07	0.07*
COVID-19 Status	0.01	-0.01	0.02	-0.01	0.01	-0.03
Perceived Infectability	0.16***	0.01	0.06	0.03	-0.04	0.04
*Psychosocial*						
Religiosity	-0.03	0.17***	0.09**	0.11***	-0.02	0.14***
Political Orientation	0.21***	0.04	0.07*	0.02	0.05	0.05
Extraversion	0.01	0.13***	0.06	0.01	-0.01	0.12***
Agreeableness	0.05	0.00	0.00	-0.04	0.04	0.00
Conscientiousness	0.13***	-0.03	0.05	-0.10**	0.09*	0.04
Neuroticism	0.09**	0.02	-0.04	-0.05	-0.01	-0.01
Openness	0.05	-0.01	-0.05	-0.06*	0.04	0.00
*COVID-19* Concern	--	0.24***	0.22***	-0.02	0.13***	0.11***
*BIS Indicators*						
Germ Aversion	0.17***	0.16***	0.21***	0.02	0.16***	0.19***
Pathogen Disgust	0.09**	0.10***	0.02	0.05	0.13***	0.11***
*R*^2^	**0.23**	**0.25**	**0.18**	**0.29**	**0.21**	**0.23**

*p < .05.

**p < .01.

***p ≤ .001. BIS = behavioral immune system; Race was coded: 1 = Not White, 0 = White. Sex was coded: 1 = female, 0 = male. Work in Healthcare was coded: 1 = yes, 0 = no. Risk Status was coded: 1 = high risk, 0 = not high risk. COVID-19 Status was coded: 1 = yes/maybe, 0 = no. Significant statistics are bold.

#### Concern about COVID-19

Overall, the model examining individual differences in concerns about COVID-19 was significant, adjusted *R*^2^ = 0.21, *F*(22, 906) = 12.35, *p* < .001. Older age, higher education, greater perceived infectability, and not having been recently ill were each significantly associated with greater COVID-19 concern. Being more liberal, greater conscientiousness, and greater neuroticism were also each significantly associated with greater concern for COVID-19. After accounting for demographic, health, social, and personality factors, greater germ aversion and greater pathogen disgust sensitivity were each significantly associated with greater concern about COVID-19.

#### Social distancing

Overall, the model examining individual differences in social distancing was significant, adjusted *R*^2^ = 0.23, *F*(23, 905) = 13.08, *p* < .001. Higher income, more recent illness, and better perceived health were each significantly associated with greater social distancing. Greater religiosity, greater extraversion, and greater COVID-19 concern were also each significantly associated with greater social distancing. After accounting for demographic, health, psychosocial, and personality factors, greater germ aversion and greater pathogen disgust sensitivity were each significantly associated with greater social distancing.

#### Avoidance of face touching

The overall model examining individual differences in avoidance of face touching was significant, adjusted *R*^2^ = 0.16, *F*(23, 927) = 8.52, *p* < .001. Greater religiosity, more liberal political ideology, and COVID-19 concern were each significantly associated with greater avoidance of touching one’s face. After accounting for demographic, health, social, and personality factors, greater germ aversion was significantly associated with avoidance of touching their face.

#### Wearing a facemask

Overall, the model examining individual differences in wearing a facemask was significant, adjusted *R*^2^ = 0.27, *F*(23, 904) = 15.69, *p* < .001. Younger age, being male, more populated location of residence, being at high risk for complications from COVID-19, not having a family member who is at high risk for complications from COVID-19, more recent illness, and better perceived health were each significantly associated with greater extent of wearing an antiviral facemask. Greater religiosity, less conscientiousness, and less openness were also each significantly associated with greater extent of wearing an antiviral facemask. After accounting for demographic, health, psychosocial, and personality factors, we did not find evidence of a significant association between any BIS indicators and wearing a facemask.

#### Handwashing

Overall, the model examining individual differences in handwashing was significant, adjusted *R*^2^ = 0.19, *F*(23, 903) = 10.33, *p* < .001. Being female and less recent illness were each significantly associated with more handwashing. Greater conscientiousness and greater COVID-19 concern were also significantly associated with more handwashing. After accounting for demographic, health, social, and personality factors, greater germ aversion and greater pathogen disgust were each significantly associated with more handwashing.

#### Cleaning/disinfecting

Overall, the model examining individual differences in cleaning or disinfecting surfaces was significant, adjusted *R*^2^ = 0.21, *F*(23, 905) = 11.51, *p* < .001. Younger age, higher income, being at high risk, not having a family member who is at high risk, more recent illness, and better perceived health were significantly associated with more cleaning/disinfecting. Additionally, greater religiosity, greater extraversion, and greater COVID-19 concern were each significantly associated with more cleaning/disinfecting. After accounting for demographic, health, social, and personality factors, greater germ aversion and greater pathogen disgust were each significantly associated with greater cleaning/disinfecting.

## Discussion

The COVID-19 pandemic represents an unprecedented infectious disease threat to people around the world. The BIS is a collection of psychological characteristics thought to serve a disease-avoidance function. The primary goal of the current study was to determine the extent to which individual differences in the BIS were uniquely associated with COVID-19 concern and COVID-19-related preventative health behavior. When demographic, health, social, personality, and BIS variables were considered simultaneously, greater germ aversion and pathogen disgust sensitivity were most consistently associated with COVID-19 concern and preventative behaviors. These findings are consistent with BIS theory and suggest potential targets for health promotion interventions.

Both germ aversion and pathogen disgust were uniquely associated with greater concern for COVID-19. Additionally, greater germ aversion was connected with greater engagement in all preventative health behaviors, except wearing a facemask, and greater pathogen disgust sensitivity was associated with more social distancing, handwashing, and cleaning/disinfecting. Notably, the effect sizes of the associations between germ aversion and preventative health behaviors were comparable to the effect sizes of the relations between COVID-19 concern and preventative health behaviors. These findings are consistent with recent work linking BIS indicators with COVID-19 related behavioral intentions and policy attitudes [14, 15]. In general, these findings support theory suggesting the protective function of psychological disease-avoidance mechanisms during a time of real disease threat [29].

The pattern of findings were relatively consistent across preventative health behaviors, except for wearing an antiviral facemask. Recommendations regarding facemasks have varied during the pandemic. At the time of data collection, the CDC only recommended wearing a facemask if individuals thought they had COVID-19, and people were dissuaded from wearing antiviral (e.g., N95) facemasks in order to prevent shortages of such personal protective equipment at hospitals and for healthcare workers [23]. On April 3, 2020 (after data collection), the CDC recommended that everyone should wear a cloth facemask in public or when around others [3]. The lack of significant associations between facemask wearing and BIS indicators or COVID-19 concern, as well as significant negative associations with other variables (e.g., conscientiousness, age), may reflect that wearing facemasks, especially antiviral facemasks, was not recommended at the time.

The present study also provided a comprehensive test of the extent to which a large range of demographic, health, and psychosocial variables were related to COVID-19 responses. Older age was associated with more concern about COVID-19, which is understandable as older adults are at higher risk of complications from COVID-19 [28]. However, this concern did not translate into greater engagement in preventative health behaviors. In fact, older age was associated with less cleaning/disinfecting behavior and utilization of antiviral facemasks. Potentially, older adults were self-isolating more and thus had less need to engage in these behaviors. Greater income was associated with more social distancing and cleaning/disinfecting behavior. Greater financial resources may facilitate the ability to engage in these practices, such as working from home and having access to disinfecting supplies. Recent illness and general perceived health were also associated with many preventative health behaviors. Well-being may be more salient for these individuals, but the underlying motivations may differ. For the former, engaging in preventative health behaviors may be motivated by wanting to protect others from getting sick. Indeed, those who had been ill recently were less concerned about COVID-19, but recent illness was the variable most strongly associated with wearing antiviral facemasks. Individuals who perceive themselves as having generally good health may be motivated to maintain their health and engage in preventative health behaviors to reduce the likelihood of getting sick themselves.

Unlike previous work [19], political orientation was not reliably associated with preventative health behavior in this nationally-representative sample. Although those who were more liberal endorsed greater concern about COVID-19, we did not find evidence that political ideology was independently associated with greater engagement in preventative health behaviors, other than greater avoidance of touching one’s face. In our study, we considered a broader range of demographic, health, and psychosocial factors than Kushner Gadarian et al. [19], which may account for the differences in results. Greater religiosity was also associated with engaging in all of the preventative health behaviors, except for handwashing. Although the empirical evidence is mixed, religiosity has been proposed to encourage prosocial behaviors [30], so these results could reflect prosociality if engaging in preventative health behaviors is for the benefit of others. Religiosity has also been associated with greater social conformity [31]. Thus, these findings may be due to religious individuals’ greater sensitivity to social norms. Consistent with the existing literature, conscientiousness and neuroticism were associated with greater concern about COVID-19. However, personality traits were not consistently related to engaging in preventative health behaviors when accounting for demographics and other psychosocial variables. In times of infectious disease threat and the unique situation of a pandemic, traditional personality traits may be a lesser determinant of behavior. Rather, personality traits may be more strongly associated with regular daily activities and lifestyle choices (e.g., physical activity, alcohol consumption).

### Implications for theory and practice

The behavioral immune system is theorized to promote the avoidance of pathogen threats. Some cross-cultural research indicates group differences in BIS reactivity due to differences in historic and contemporary parasite or disease prevalence rates associated with geographic locations [32, 33]. However, most of the BIS research has been conducted in lab-based settings using artificial tasks designed to prompt disease threat [22]. The current study extends this body of evidence and further supports BIS theory by demonstrating that individual differences in germ aversion and pathogen disgust sensitivity were associated with responses to a real-world, contemporary pathogen threat. Such an extension provides important ecological validity to lab-based and historical research.

Findings from this study have implications for policy and public response to future epidemics or pandemics. Our results identify a variety of demographic and psychosocial characteristics that may place individuals at-risk for contracting and spreading disease during pandemics. Designing efficacious messaging that targets these populations may help optimize alterations in human behavior necessary to prevent the spread of infectious diseases. For instance, in addition to focusing on the severity of infection, it may be beneficial for messages about COVID-19 to emphasize aspects of the virus and disease that activate the psychological disease-avoidance processes. For example, messages could incorporate images or language that induce feelings of disgust or make salient the presence of germs or contamination. Although individuals reliably differ in these psychological traits, there are malleable components to the disease-avoidance processes that can be activated, leading to behavior change [29]. Thus, it may be important for future research to examine whether temporarily increasing disgust or germ aversion promotes pandemic-related health behaviors.

### Limitations

Results from this study should be taken in light of certain limitations. The data are cross-sectional preventing causal claims; however, the wide range of demographic and psychosocial variables considered reduces the possibility of third variables. Of course, other variables not assessed in the current study may still account for the findings. Theoretically, germ aversion and pathogen disgust sensitivity motivate concern about infectious disease and engagement in preventative health behaviors. Experimental evidence indicates that inducing disgust results in greater avoidance behavior, reducing potential contact with pathogens [34]. However, we cannot rule out the opposite causal relation with the current data. Greater concern about COVID-19 may lead to increased germ aversion and pathogen disgust sensitivity. Experimental or longitudinal data are necessary to determine the causal direction. All variables were assessed with self-report measures, which raises concerns of social desirability or biased responding. At the time of data collection, there were no publically available measures of COVID-19 concern, so we developed our own measure. Although reliable and the items were deemed to be face valid by the research group, the COVID-19 concern measure was not validated. Also, data were collected online. Although a nationally representative sample was recruited, only individuals with reliable internet access, computer, or smartphone were able to complete the study, thus limiting generalizability.

## Conclusion

Our study has highlighted several factors associated with concern about COVID-19 and engagement in preventative health behaviors. Further research is needed to test these associations prospectively. These findings identify those who may be at greater risk of contracting and spreading COVID-19, or future infectious diseases, as well as provide potential targets for interventions intended to increase preventative health behaviors to reduce pathogen transmission.

## Supporting information

S1 Table Regression model for COVID-19 concern. (DOCX)

S2 Table Regression model for social distancing. (DOCX)

S3 Table Regression model for avoid touching face. (DOCX)

S4 Table Regression model for wearing antiviral facemask. (DOCX)

S5 Table Regression model for handwashing. (DOCX)

S6 Table Regression model for cleaning/disinfecting. (DOCX)

S1 Appendix Study measures. (DOCX)
