# Supplementary material for: Disease avoidance in the time of COVID-19: The behavioral immune system is associated with concern and preventative health behaviors
Source: PLoS One. 2020 Aug 20;15(8):e0238015. doi: 10.1371/journal.pone.0238015 (PMC7446877; doi:10.1371/journal.pone.0238015)
Supplement: S3 Table — (DOCX) [file pone.0238015.s003.docx]

**S3 Table. Regression model with avoid touching face as the outcome**

|  | Avoid Face Touching | | | |
| --- | --- | --- | --- | --- |
|  | *B* | 95% CI | *SE* | *β* |
| *Demographics* |  |  |  |  |
| Age | -0.01 | [-0.02,0.00] | 0.00 | -0.07 |
| Race | 0.11 | [-0.19,0.41] | 0.15 | 0.02 |
| Sex | 0.07 | [-0.17,0.32] | 0.12 | 0.02 |
| Education | 0.05 | [-0.03,0.12] | 0.04 | 0.04 |
| Income | 0.03 | [-0.01,0.07] | 0.02 | 0.05 |
| Hometown | 0.04 | [-0.03,0.12] | 0.04 | 0.04 |
| Work in Healthcare | 0.20 | [-0.23,0.62] | 0.22 | 0.03 |
| Risk Status (Self) | -0.11 | [-0.38,0.15] | 0.13 | -0.03 |
| Risk Status (Family) | 0.09 | [-0.16,0.34] | 0.13 | 0.02 |
| Illness Recency | 0.06 | [-0.03,0.15] | 0.05 | 0.05 |
| Perceived Health | 0.12 | [-0.03,0.26] | 0.08 | 0.05 |
| COVID-19 Status | 0.11 | [-0.22,0.43] | 0.17 | 0.02 |
| *Psychosocial* |  |  |  |  |
| Religiosity | 0.05 | [0.01,0.09] | 0.02 | **0.09**** |
| Political Orientation | 0.12 | [0.01,0.24] | 0.06 | **0.07*** |
| Extraversion | 0.12 | [-0.01,0.24] | 0.07 | 0.06 |
| Agreeableness | -0.01 | [-0.15,0.14] | 0.07 | 0.00 |
| Conscientiousness | 0.10 | [-0.06,0.26] | 0.08 | 0.05 |
| Neuroticism | -0.07 | [-0.21,0.07] | 0.07 | -0.04 |
| Openness | -0.12 | [-0.26,0.02] | 0.07 | -0.05 |
| COVID-19 Concern | 0.60 | [0.42,0.79] | 0.09 | **0.22***** |
| *Disease Avoidance* |  |  |  |  |
| Perceived Infectability | 0.11 | [-0.02,0.23] | 0.06 | 0.06 |
| Germ Aversion | 0.42 | [0.29,0.56] | 0.07 | **0.21***** |
| Pathogen Disgust | 0.03 | [-0.08,0.15] | 0.06 | 0.02 |
| *R*^2^ | **0.18** | | | |

*Note*. **p* < .05. ***p* < .01. ****p* ≤ .001. Race was coded: 1 = Not White, 0 = White. Sex was coded: 1 = Female, 0 = Male. Work in Healthcare was coded: 1 = yes, 0 = no. Risk Status was coded: 1 = high risk, 0 = not high risk. COVID-19 Status was coded: 1 = yes/maybe, 0 = no. Significant statistics are bold.
