# Supplementary material for: Disease avoidance in the time of COVID-19: The behavioral immune system is associated with concern and preventative health behaviors
Source: PLoS One. 2020 Aug 20;15(8):e0238015. doi: 10.1371/journal.pone.0238015 (PMC7446877; doi:10.1371/journal.pone.0238015)
Supplement: S4 Table — (DOCX) [file pone.0238015.s004.docx]

**S4 Table. Regression model with wearing antiviral facemask as the outcome**

|  | Wearing Antiviral Facemask | | | |
| --- | --- | --- | --- | --- |
|  | *B* | 95% CI | *SE* | *β* |
| *Demographics* |  |  |  |  |
| Age | -0.01 | [-0.02,0.00] | 0.00 | **-0.11**** |
| Race | 0.03 | [-0.17,0.23] | 0.10 | 0.01 |
| Sex | -0.28 | [-0.44,-0.12] | 0.08 | **-0.10***** |
| Education | -0.02 | [-0.07,0.03] | 0.03 | -0.03 |
| Income | 0.02 | [-0.01,0.05] | 0.01 | 0.05 |
| Hometown | 0.07 | [0.02,0.12] | 0.03 | **0.08*** |
| Work in Healthcare | 0.22 | [-0.06,0.51] | 0.14 | 0.05 |
| Risk Status (Self) | 0.31 | [0.13,0.48] | 0.09 | **0.11***** |
| Risk Status (Family) | -0.17 | [-0.33,0.00] | 0.08 | **-0.06*** |
| Illness Recency | 0.26 | [0.20,0.32] | 0.03 | **0.31***** |
| Perceived Health | 0.19 | [0.10,0.29] | 0.05 | **0.13***** |
| COVID-19 Status | -0.03 | [-0.25,0.19] | 0.11 | -0.01 |
| *Psychosocial* |  |  |  |  |
| Religiosity | 0.04 | [0.02,0.07] | 0.01 | **0.11***** |
| Political Orientation | 0.02 | [-0.05,0.10] | 0.04 | 0.02 |
| Extraversion | 0.02 | [-0.07,0.10] | 0.04 | 0.01 |
| Agreeableness | -0.06 | [-0.15,0.04] | 0.05 | -0.04 |
| Conscientiousness | -0.16 | [-0.26,-0.05] | 0.05 | **-0.10**** |
| Neuroticism | -0.07 | [-0.16,0.03] | 0.05 | -0.05 |
| Openness | -0.10 | [-0.19,0.00] | 0.05 | **-0.06*** |
| COVID-19 Concern | -0.04 | [-0.16,0.09] | 0.06 | -0.02 |
| *Disease Avoidance* |  |  |  |  |
| Perceived Infectability | 0.04 | [-0.04,0.12] | 0.04 | 0.03 |
| Germ Aversion | 0.03 | [-0.06,0.12] | 0.05 | 0.02 |
| Pathogen Disgust | 0.06 | [-0.01,0.14] | 0.04 | 0.05 |
| *R*^2^ | 0.29 | | | |

*Note*. **p* < .05. ***p* < .01. ****p* ≤ .001. Race was coded: 1 = Not White, 0 = White. Sex was coded: 1 = Female, 0 = Male. Work in Healthcare was coded: 1 = yes, 0 = no. Risk Status was coded: 1 = high risk, 0 = not high risk. COVID-19 Status was coded: 1 = yes/maybe, 0 = no. Significant statistics are bold.
