# Supplementary material for: Disease avoidance in the time of COVID-19: The behavioral immune system is associated with concern and preventative health behaviors
Source: PLoS One. 2020 Aug 20;15(8):e0238015. doi: 10.1371/journal.pone.0238015 (PMC7446877; doi:10.1371/journal.pone.0238015)
Supplement: S5 Table — (DOCX) [file pone.0238015.s005.docx]

**S5 Table. Regression model with handwashing as the outcome**

|  | Handwashing | | | |
| --- | --- | --- | --- | --- |
|  | *B* | 95% CI | *SE* | *β* |
| *Demographics* |  |  |  |  |
| Age | 0.00 | [-0.01,0.01] | 0.00 | 0.00 |
| Race | -0.16 | [-0.4,0.07] | 0.12 | -0.04 |
| Sex | 0.33 | [0.13,0.52] | 0.10 | **0.10***** |
| Education | -0.04 | [-0.1,0.02] | 0.03 | -0.04 |
| Income | 0.01 | [-0.02,0.05] | 0.02 | 0.03 |
| Hometown | 0.03 | [-0.03,0.09] | 0.03 | 0.03 |
| Work in Healthcare | 0.10 | [-0.23,0.44] | 0.17 | 0.02 |
| Risk Status (Self) | 0.14 | [-0.07,0.35] | 0.11 | 0.05 |
| Risk Status (Family) | 0.10 | [-0.1,0.29] | 0.10 | 0.03 |
| Illness Recency | -0.12 | [-0.19,-0.05] | 0.04 | **-0.12***** |
| Perceived Health | 0.11 | [0,0.23] | 0.06 | 0.07 |
| COVID-19 Status | 0.03 | [-0.23,0.29] | 0.13 | 0.01 |
| *Psychosocial* |  |  |  |  |
| Religiosity | -0.01 | [-0.04,0.02] | 0.02 | -0.02 |
| Political Orientation | 0.07 | [-0.02,0.16] | 0.05 | 0.05 |
| Extraversion | -0.01 | [-0.12,0.09] | 0.05 | -0.01 |
| Agreeableness | 0.07 | [-0.05,0.19] | 0.06 | 0.04 |
| Conscientiousness | 0.15 | [0.02,0.28] | 0.07 | **0.09*** |
| Neuroticism | -0.01 | [-0.12,0.1] | 0.06 | -0.01 |
| Openness | 0.07 | [-0.04,0.18] | 0.06 | 0.04 |
| COVID-19 Concern | 0.30 | [0.15,0.44] | 0.07 | **0.13***** |
| *Disease Avoidance* |  |  |  |  |
| Perceived Infectability | -0.06 | [-0.16,0.05] | 0.05 | -0.04 |
| Germ Aversion | 0.27 | [0.16,0.37] | 0.05 | **0.16***** |
| Pathogen Disgust | 0.19 | [0.1,0.28] | 0.05 | **0.13***** |
| *R*^2^ | **0.21** | | | |

*Note*. **p* < .05. ***p* < .01. ****p* ≤ .001. Race was coded: 1 = Not White, 0 = White. Sex was coded: 1 = Female, 0 = Male. Work in Healthcare was coded: 1 = yes, 0 = no. Risk Status was coded: 1 = high risk, 0 = not high risk. COVID-19 Status was coded: 1 = yes/maybe, 0 = no. Significant statistics are bold.
