# Supplementary material for: Disease avoidance in the time of COVID-19: The behavioral immune system is associated with concern and preventative health behaviors
Source: PLoS One. 2020 Aug 20;15(8):e0238015. doi: 10.1371/journal.pone.0238015 (PMC7446877; doi:10.1371/journal.pone.0238015)
Supplement: S6 Table — (DOCX) [file pone.0238015.s006.docx]

**S6 Table. Regression model with cleaning/disinfecting as the outcome**

|  | Cleaning/Disinfecting | | | |
| --- | --- | --- | --- | --- |
|  | *B* | 95% CI | *SE* | *β* |
| *Demographics* |  |  |  |  |
| Age | -0.01 | [-0.02,-0.01] | 0.00 | **-0.14***** |
| Race | 0.05 | [-0.17,0.27] | 0.11 | 0.01 |
| Sex | -0.03 | [-0.21,0.16] | 0.09 | -0.01 |
| Education | -0.05 | [-0.11,0] | 0.03 | -0.06 |
| Income | 0.03 | [0,0.06] | 0.02 | **0.07*** |
| Hometown | 0.02 | [-0.03,0.08] | 0.03 | 0.02 |
| Work in Healthcare | 0.05 | [-0.27,0.36] | 0.16 | 0.01 |
| Risk Status (Self) | 0.23 | [0.03,0.43] | 0.10 | **0.08*** |
| Risk Status (Family) | -0.30 | [-0.49,-0.12] | 0.09 | **-0.10***** |
| Illness Recency | 0.17 | [0.11,0.24] | 0.03 | **0.19***** |
| Perceived Health | 0.12 | [0.01,0.23] | 0.06 | **0.07*** |
| COVID-19 Status | -0.11 | [-0.35,0.14] | 0.12 | -0.03 |
| *Psychosocial* |  |  |  |  |
| Religiosity | 0.06 | [0.03,0.09] | 0.01 | **0.14***** |
| Political Orientation | 0.07 | [-0.02,0.15] | 0.04 | 0.05 |
| Extraversion | 0.18 | [0.09,0.28] | 0.05 | **0.12***** |
| Agreeableness | 0.00 | [-0.11,0.11] | 0.06 | 0.00 |
| Conscientiousness | 0.07 | [-0.05,0.19] | 0.06 | 0.04 |
| Neuroticism | -0.01 | [-0.12,0.09] | 0.05 | -0.01 |
| Openness | -0.01 | [-0.11,0.1] | 0.05 | 0.00 |
| COVID-19 Concern | 0.23 | [0.09,0.36] | 0.07 | **0.11***** |
| *Disease Avoidance* |  |  |  |  |
| Perceived Infectability | 0.05 | [-0.05,0.14] | 0.05 | 0.04 |
| Germ Aversion | 0.30 | [0.2,0.4] | 0.05 | **0.19***** |
| Pathogen Disgust | 0.15 | [0.07,0.24] | 0.04 | **0.11***** |
| *R*^2^ | **0.23** | | | |

*Note*. **p* < .05. ***p* < .01. ****p* ≤ .001. Race was coded: 1 = Not White, 0 = White. Sex was coded: 1 = Female, 0 = Male. Work in Healthcare was coded: 1 = yes, 0 = no. Risk Status was coded: 1 = high risk, 0 = not high risk. COVID-19 Status was coded: 1 = yes/maybe, 0 = no. Significant statistics are bold.
