# Supplementary material for: Disease avoidance in the time of COVID-19: The behavioral immune system is associated with concern and preventative health behaviors
Source: PLoS One. 2020 Aug 20;15(8):e0238015. doi: 10.1371/journal.pone.0238015 (PMC7446877; doi:10.1371/journal.pone.0238015)
Supplement: S1 Appendix — (DOCX) [file pone.0238015.s007.docx]

**S1 Appendix. Study measures**

**10-item Big Five Inventory (Rammstedt & John, 2007)**

How well do the following statements describe your personality? I see myself as someone who....

- Disagree Strongly
- Disagree a little
- Neither Agree or disagree
- Agree a little
- Agree Strongly

1. is reserved.
2. is generally trusting.
3. tends to be lazy.
4. is relaxed, handles stress well.
5. has few artistic interests
6. is outgoing, sociable.
7. tends to find the fault with others.
8. does a thorough job.
9. gets nervous easily.
10. has an active imagination

**Perceived Vulnerability to Disease Questionnaire** **(Duncan, Schaller, & Park, 2009)**

Please indicate the extent to which you agree or disagree with each statement below using the following scale.  There are no right or wrong answers. Please think about each statement carefully before answering.

- Strongly Disagree
- Disagree
- Slightly Disagree
- Neither agree nor disagree
- Slightly Agree
- Agree
- Strongly Agree

1.     In general, I am very susceptible to colds, flu and other infectious diseases.

2.     I am unlikely to catch a cold, flu or other illness, even if it is ‘going around’.

3.     If an illness is ‘going around’, I will get it.

4.     My immune system protects me from most illnesses that other people get.

5.     I am more likely than the people around me to catch an infectious disease.

6.     My past experiences make me believe I am not likely to get sick even when my friends are sick.

7.     I have a history of susceptibility to infectious disease.

8.     I prefer to wash my hands pretty soon after shaking someone’s hand.

9.     I avoid using public telephones because of the risk that I may catch something from the previous user.

10.  I do not like to write with a pencil someone else has obviously chewed on.

11.  I dislike wearing used clothes because you do not know what the last person who wore it was like.

12.  I am comfortable sharing a water bottle with a friend.

13.  It really bothers me when people sneeze without covering their mouths.

14.  It does not make me anxious to be around sick people.

15.  My hands do not feel dirty after touching money.

**Pathogen Disgust Subscale (Tybur, Lieberman, & Griskevicius, 2009)**

The following items describe a variety of concepts. Please rate how disgusting you find the concepts described in the items.

0 1 2 3 4 5 6

Not disgusting Extremely

at all disgusting

1. Stepping on dog poop
2. Sitting next to someone who has red sores on their arm
3. Shaking hands with a stranger who has sweaty palms
4. Seeing some mold on old leftovers in your refrigerator
5. Standing close to a person who has body odor
6. Seeing a cockroach run across the floor
7. Accidentally touching a person’s bloody cut

**COVID-19 Concern**

How concerned are you about the coronavirus, COVID-19?

- Very concerned
- Somewhat concerned
- Not sure
- Not too concerned
- Not at all concerned

How strongly do you agree or disagree with each statement?

- Strongly Disagree
- Disagree
- Slightly Disagree
- Neither Agree nor Disagree
- Slightly Agree
- Agree
- Strongly Agree

1. The coronavirus is just the flu or a common cold
2. People are greatly exaggerating the threat of the coronavirus
3. The coronavirus is as serious as people make it out to be
4. The coronavirus has been blown out of proportion
5. People are not doing enough to prevent the spread of the coronavirus

**COVID-19 Infection Status**

Do you think that you currently have or previously had COVID-19?

- Yes
- Maybe
- No

**COVID-19 Preventative Behaviors**

To what extent are you engaging in social distancing (i.e., reducing contact with others to avoid contracting COVID-19)?

- A great deal
- A lot
- Somewhat
- A little
- Not at all

In the **past 7 days**, how often did you:

- Not at all
- Once
- Twice
- 3-4 times
- 5-6 times
- Daily
- Multiple times a day

1. Avoid shaking someone’s hand for greeting
2. Avoid hugging someone for greeting
3. Avoid kissing someone for greeting
4. Avoid going to school/job
5. Order food online or get take out rather eating at a restaurant
6. Avoid touching your face.
7. Wear an antiviral facemask
8. Carry anti-bacterial hand sanitizer with you throughout the day.
9. Wash hands for at least 20 seconds
10. Clean and disinfect surfaces in your home with antibacterial wipes.
11. Clean your mobile phone
12. Clean your laptop

**Perceived Health**

**In general**, would you say your health is:

1= excellent

2= very good

3 = good

4 = fair

5 = poor

**Illness Recency (Miller & Maner, 2011)**

Please indicate your agreement with four statements:

1 2 3 4 5 6 7

Strongly Strongly

disagree agree

1.     Over the past couple days, I have not been feeling well.

2.     Lately, I have been feeling a little under the weather.

3.     I have felt sick within the past week.

4.     I had a cold or flu recently.

**Family Health History Questions**

Please give information on the medical history of you and any blood relatives. Please indicate whether you or a family member (e.g. your mother, father, sister, brother, aunt, uncle, etc.) have or previously had any of the following conditions. If information is unknown (“unk”) or not available (“N/A”), please indicate.

**Gastrointestinal**

Ulcers

Inflammatory Bowel

Cleft Lip or Palate

Other

**Cardiovascular**

High Blood Pressure

Heart Attack

Stroke

Congestive Heart Failure

Atherosclerosis

Heart Rhythm Abnormality

Congenital Heart Defect

**Immune/Hematological Condition**

Mononucleosis

Hemophilia

Leukemia

Lymphomas

Hodgkin’s Disease

Factor V Leiden

**Renal Condition**

Kidney Failure

Dialysis/Transplant

Other Kidney

**Liver Disease**

Hepatitis (specify)

Cirrhosis

Other Liver Disease

**Central Nervous System Condition**

Epilepsy

Hydrocephalus

Multiple Sclerosis

Huntington’s Chorea

Seizures/convulsions

**Endocrine**

Diabetes (adult or juvenile)

Thyroid (Hyper/Hypo)

Adrenal

**Muscular/Skeletal**

Club Foot

Scoliosis

Arthritis (Osteo or Rheumatoid)

Lupus

**Neuromuscular**

Cerebral Palsy

Muscular Dystrophy

Spina Bifida

**Visual/Auditory**

Blindness

Glaucoma

Cataracts

Deafness or Other Hearing Problems

**Other Conditions**

Mental Illness (e.g. Depression, Bipolar, Schizophrenia)

Alcohol or Drug Abuse

Eating Disorders

Mental Retardation or Developmental Disability

**Political Orientation**

What is your political orientation?

• Very conservative

• Conservative

• Moderate

• Liberal

• Very liberal

**Religiosity**

How religious are you, in general? Please select the number that best describes your experience, with 0 indicating the minimum and 10 indicating higher religiosity:

0 1 2 3 4 5 6 7 8 10

Not at all religious Extremely religious

**Demographics**

Age:

What sex were you assigned at birth?    Male        Female       Other_______

Race/Ethnicity (check all that apply):

                                    White/Caucasian

                                    Hispanic/Latino(a)

                                    African-American/Black

                                    Asian

                                    Native American

                                    Other (Please indicate):

What would you estimate your combined family income to be?

• Less than $10,000

• $10,000 - $19,999

• $20,000 - $29,999

• $30,000 - $39,999

• $40,000 - $49,999

• $50,000 - $59,999

• $60,000 - $69,999

• $70,000 - $79,999

• $80,000 - $89,999

• $90,000 - $99,999

• $100,000 - $149,999

• More than $150,000

Education:

• Less than/some high school

• GED/high school equivalency

• High school graduate

• Vocation/trade school

• Some college

• Associate’s 2-year degree

• College graduate

• Graduate studies/degree

How would you characterize your hometown?

• Rural (unincorporated)

• Small town (village or town)

• Suburban (metropolitan area of a large city)

• Small city (population <30,000)

• Medium-sized city (population 30,000 to 100,000)

• Large city (population >100,000)

Do you work in the healthcare field (e.g., physician, nurse, medical assistant, pharmacist, etc.)?

• Yes

• No

For females, are you currently pregnant?

• Yes

• Maybe

• No

Are you taking any medications that suppress your immune system?

• Yes

• Maybe

• No
